# A Case of Pregnancy Which Terminated Fatally, with an Account of an Unusual Appearanee of the Uterus

**Published:** 1803-05-01

**Authors:** 


*A* CASE *of* PREGNANCY *which terminated fatally, with an Account of an unusual Appearanee of the Uterus, by Cit.* NEYRONIS. *Communicated by our Correspondent at Paris.*

THE author tells us that he was called to a woman aged 42, who was then pregnant of her thirteenth child. The account she gave of herself was, that at the fifth month she had some flooding which lasted four days, accompanied by very sharp pains in the loins and cramps in the extremities. These complaints returned again on the seventh month. When Cit. N. was called she laboured under a third attack, more severe than the preceding, and from the strength and nature of the pains he had reason to expect a speedy delivery. He had remarked, however, that there was not the least appearance of dilatation of the os tincæ. She complained much of a sense of weight towards towards the orifice of the womb, and which she referred to the right side when she lay on the right, and vice versa. On questioning her more particularly, she said, that she had not perceived the child stir for five days; the pains, losses, and other circumstances just described had continued four days; the waters had been discharged since the first. From the resistance which the matrix offered, he thought it necessary to bleed her twice; many clysters and baths were had recourse to, without any advantage whatever. The pains ceased suddenly, and the patient very soon expired. The author tells us that he proceeded immediately to open the body, when the following appearances presented themselves: On cutting through the integuments, muscles, &c. into the uterus he found a male child, the head of which was of a monstrous size without being dropsical, measuring 22 inches in circumference; the epidermis came off on the least touch. He found the matrix three inches thick towards its fundus, particularly where it lies towards the bladder; but what most surprised him was, that its thickness at the neck was not so considerable, and which was nearly in its natural state. This thickness, or rather carcinomatous appearance, the author asks, was it not the cause of the floodings as well of preventing the pains from affecting the neck of the uterus so as to produce dilatation? What was to be done, he asks, to remedy a similar defect? Ought the Cæsarean operation be resorted to when every circumstance showed the child to be dead? Lastly, he asks, was it advisable or practicable when it was clear that the uterus itself made no efforts to discharge its contents? These questions the author puts without attempting to solve them, and contents himself with proposing the facts.

